# An osteometric and 3D analysis of the atlanto‐occipital joint: An initial screening method to exclude crania and atlases in commingled remains

**DOI:** 10.1002/ajpa.24437

**Published:** 2021-11-09

**Authors:** Annalisa Cappella, Luciana Affatato, Daniele Gibelli, Debora Mazzarelli, Matteo Zago, Claudia Dolci, Chiarella Sforza, Cristina Cattaneo

**Affiliations:** ^1^ Dipartimento di Scienze Biomediche per la Salute Università degli Studi di Milano Milan; ^2^ U.O. Laboratorio di Morfologia Umana Applicata IRCCS Policlinico San Donato San Donato Milanese MI; ^3^ Laboratorio di Antropologia e Odontologia Forense, Sezione Medicina Legale, Dipartimento di Scienze Biomediche per la Salute Università degli Studi di Milano Milan Italy; ^4^ Dipartimento di meccanica Politecnico di Milano Milan Italy

**Keywords:** 3D analysis, anatomical variability, Atlanto‐occipital joint, commingled remains, osteometrics

## Abstract

**Objectives:**

The anatomical features of the atlanto‐occipital joint can be potentially useful in re‐associating or excluding crania to atlases in commingled remains. This study investigated whether linear measurements and the 3‐dimensional (3D) surface of occipital condyles and articular facets of atlases can represent valid insights for this purpose.

**Methods:**

The variations among eight corresponding linear distances were analyzed in a sample of 150 individuals through six supervised machine learning techniques attempting to develop classifiers able to identify elements belonging to the same individual. Furthermore, a 3D analysis was conducted on the articular surfaces through superimpositions of 3D models of corresponding and non‐corresponding crania and atlases obtained by using respectively stereophotogrammetry and laser scanning. This analysis investigated differences in terms of point‐to‐point distances (Root Mean Square, RMS) of superimposed 3D surfaces.

**Results:**

None of the six machine learning techniques were able to correctly detect a satisfying percentage of correspondent pairs in the overall sample by using the linear variables. The 3D analysis of the articular surfaces found RMS values over 0.53 mm only for superimposed non‐corresponding surfaces, which sets a threshold value to identify 32% of incorrect pairs.

**Discussion:**

The re‐association of cranium to atlas proved to be challenging and hardly possible when considering only metric variables. However, the 3D geometry of the articular surfaces represents a valid variable for this purpose and 3D analyses pave the way for an initial exclusion of incorrect re‐associations, thus should not be considered as a re‐association method per se, but as an exclusionary screening technique.

## Graphical abstract

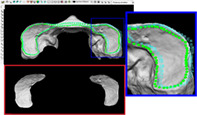

## 1 INTRODUCTION

Commingled human remains refers to the mixing of skeletal remains of two or more individuals, regardless of cause or context. The commingling can be limited to few subjects, as in the case of graves of multiple individuals or in fatal motor vehicle accidents, or it can be extended to a large number of individuals, as in cases of mass graves or mass disasters. Whether such contexts pertain to archeological or forensic scenarios or comprise a few or hundreds of individuals, the aim of anthropologists is the re‐association of the entire skeleton of each individual in order to reconstruct their story and identity.

While numerous methods have been developed, the decision of which method to use for handling commingled human remains depends largely upon the situation and the objectives of the anthropologists. Sorting techniques are very useful to re‐associate remains in archeological and forensic contexts and can be classified as “sorting based on osteometry” and “sorting based on morphological features”.

Morphological techniques consist of segregating bones by age at death and sex using established methods, rejoining complementary fragmentary elements, and individualization by weight/mass of long bones, robusticity, muscle markings, symmetry, and evidence of pathological conditions (Adams & Byrd, 2005; Adams & Konigsberg, 2004, 2008; Baker & Newman, 1957; Byrd & LeGarde, 2018; Garrido‐Varas et al., 2015; Lynch et al., 2018; Nikita & Lahr, 2011; Snow & Folk, 1965; Ubelaker, 2002; Vehit & Christensen, 2019). Osteometric techniques measure bone sizes and create regression models based on linear relationships between bones in order to compare their similarity with the final purpose to characterize referenced normal size and relationships among bone elements (Byrd, 2014). This is accomplished using estimates of population parameters (mean and standard deviation (SD)) from reference data, used to formulate the statistical hypothesis to be subjected to a significance test (Byrd, 2014; Fisher, 1958).

There are three basic approaches to osteometric sorting: comparison of the left and right bones using reference models (paired elements), comparison of adjoining bones with models that demonstrates correlation between joint surfaces (articulating bones), and comparison of the bone size with the use of regression models.

Morphological and metric assessments might be supported also by 3D technologies. These technologies could help researchers to investigate, organize and document commingled remains, but they are also able to quantify compatibility, similarity and matching in order to aid the sorting and comparison of bone elements (Anastopoulou et al., 2019). In recent years, some of the current technologies available, such as Conventional Radiology, Computed Tomography (CT), Magnetic Resonance Imaging (MRI), laser scanning and stereophotogrammetry were successfully adopted in general forensic investigations, but also in contexts of mass disasters (Bisset et al., 2002; de Jong et al., 2020; Lynnerup et al., 2017; O'Donnell et al., 2011; Viner, 2014). These technologies are noninvasive and provide the quick acquisition of accurate and reliable images, which can then be used to study and repeatedly reconstruct bones without altering the original structure, and generate computerized methods to collect osteometric data and 3D virtual models (Stull et al., 2014). Recently, a few studies used 3D approaches for validating new next‐generation sorting methods for osteological pair‐matching (Karell et al., 2016; Fancourt et al., 2021). Nevertheless, their potential use in the re‐association of corresponding bone elements at their joints has not yet been investigated.

Overall, except for the numerous studies focusing on sorting pair‐matches, the literature still shows a scarcity of methods based on the re‐association of specific joint complexes to the level of the individual. Buikstra and Gordon (1980) have focused on adjacent cervical vertebrae; two studies have taken bones of the hip joint into consideration (London & Curran, 1986; London & Hunt, 1998); recently articulating lower limb bones have been surveyed (Anastopoulou et al., 2018, 2019), and a recent study examined the compatibility of match and mismatch CT models of mandibles and crania through a 3D approach (Preissler et al., 2018). Nevertheless, many articulations still need to be examined for re‐association. The atlanto‐occipital joint is one that has not been assessed despite its potential crucial role in re‐associating the entire body.

The current study focused on the specific problem of re‐associating small scale commingling involving the cranium and the remaining and yet articulated body. More specifically, this study focuses on the problem of re‐associating the occipital condyles to the atlas of an articulating body lacking the cranium. This situation was found to be frequent in recent humanitarian disasters (Cattaneo et al., 2020; Piscitelli et al., 2016) of the Mediterranean. Such a situation includes archeological scenarios where burials with multiple individuals are commingled, with an emphasis on disassociated crania, such as can be found in necropolises, ossuaries and crypts (Adams & Byrd, 2014; Duday, 2009).

To the best of our knowledge, to date only one study has focused on the quantification of 2D and 3D anatomical variation at the atlanto‐occipital articulation with the intent of providing reference standards for the re‐association of individuals. However, Dudar and Castillo (2016) focused mainly on differences in size and shape associated with sexual dimorphism and biological ancestry, as well as the 3D biomechanical variations and congruency of condylar angle and articular surface, but without providing any practical method to classify elements belonging to the same individual. Thus, no comprehensive study has focused on the re‐association of crania to the post‐crania (specifically to the atlas) with the intent to provide a mathematical and statistical method to classify elements belonging to the same individual. In particular, no study has used a 3D approach enabling the superimposition between corresponding articular surfaces and by developing statistical quantifying tools able to discriminate among correct and incorrect matches.

The present study aims to investigate whether osteometry (direct linear distances) and 3D analysis of surfaces (3D point‐to‐point distances superimposition) might prove to be valid for re‐associating the cranium to the corresponding atlas. In particular, the osteometric and 3D articular surface variability of the occipital bone (condyles) and atlas (superior articular facets) were examined in order to investigate if these quantitative variables are useful for re‐associating the cranium and atlas.

## 2 MATERIALS AND METHODS

### 2.1 Linear osteometric survey

#### 2.1.1 Sample and data collection

This observational study examined crania and their associated first cervical vertebrae of known adult individuals from a cemetery skeletal collection (CAL, Collezione Antropologica Labanof) (Biehler‐Gomez et al., 2018; Castoldi et al., 2018; Cattaneo et al., 2018) housed at the University of Milan. The sample consists of 150 atlanto‐occipital bones selected from 83 male and 67 female individuals with an age range of 19–93 years. The study sample was selected from on a total sample of 300 individuals according to three main criteria: (i) the presence of the two bones (occipital bone and atlas); (ii) the perfect state of preservation of the two bones (without taphonomic or traumatic alterations); iii) the absence of any pathological condition such as osteoarthritis, metastases etc., which might have modified both morphology and dimensions.

**FIGURE 1:**
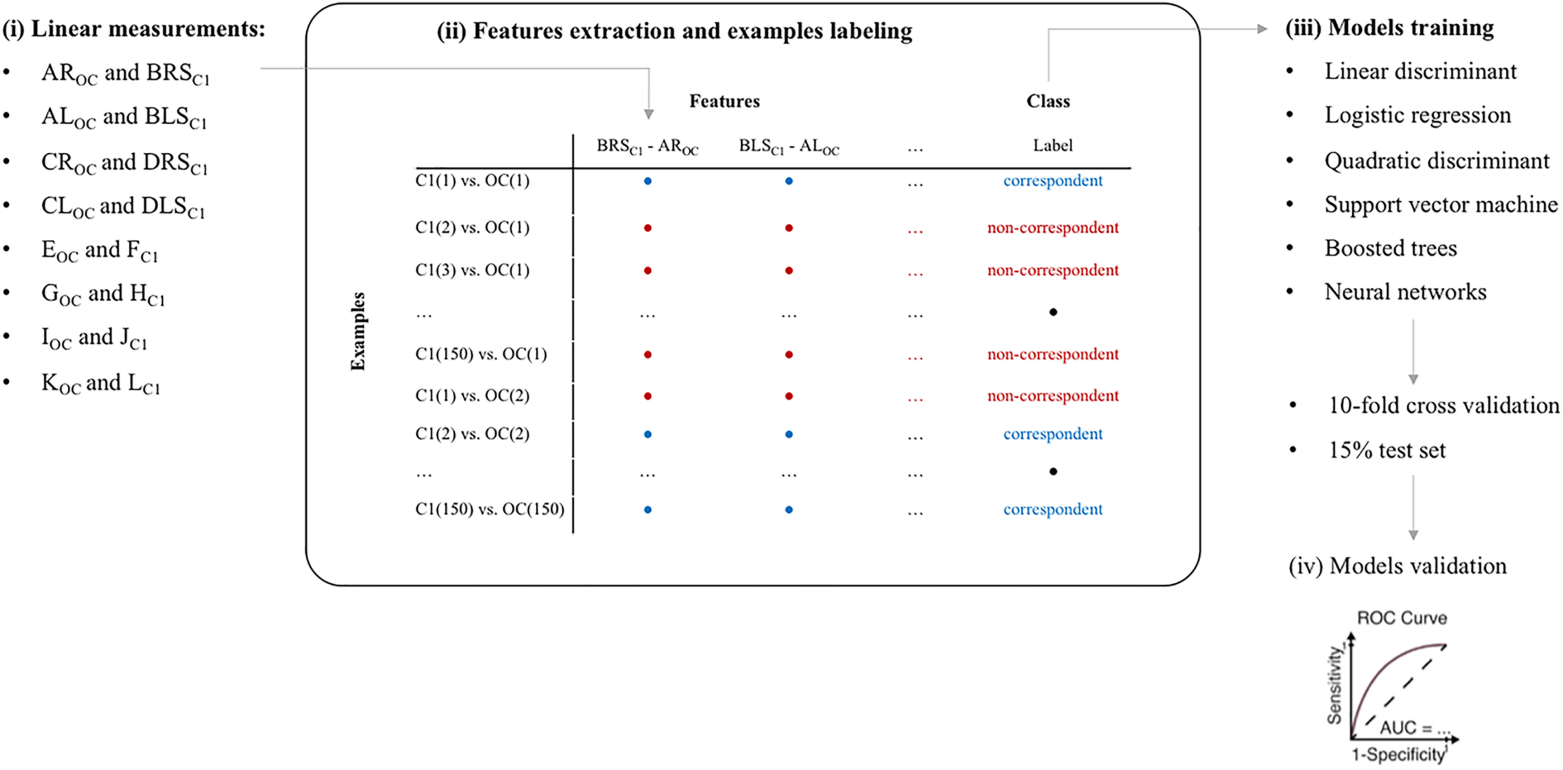
Data processing flow, including the four main steps: (i) linear measurements, (ii) features extraction, (iii) models training, and (iv) models validation. See Table 1 for definitions

A total of 16 linear osteometric measurements were taken on the occipital bone and atlas (Figure 1) as previously described by Dudar and Castillo (2016) by means of a manual sliding caliper (resolution of 0.05 mm). Eight measurements were conducted on the inferior aspect of the occipital and eight on the superior aspect of the atlas. Each of these measurements corresponds to the other based on mechanical matching: ex. maximum length of the left occipital condyle and maximum length of the left superior articular facet of the atlas (Table 1). These measurement pairs were chosen to test the potential osteometric correspondence between the occipital and atlas for the same individual (namely ‘associated bones’). In other words, to determine if osteometric differences detectable from associated/matching occipital‐atlas measurements are a sufficient variable to discriminate between elements originating from the same individual or from different ones. This aim was subsequently investigated through an in‐depth statistical approach.

**TABLE 1:** List of occipital and atlas measurements with related definitions and abbreviations

Occipital bone		Atlas	
Measurement abbreviation	Definition	Measurement abbreviation	Definition
AR_OC_	Measured from the medial point to the lateral point of the widest area of the right occipital condyle	BRS_C1_	Measured from the medial point on the widest spot of the atlas right articular facet to the lateral point on the widest spot of the same articular facet
AL_OC_	Measured from the medial point to the lateral point of the widest area of the left occipital condyle	BLS_C1_	Measured from the medial point on the widest spot of the atlas left articular facet to the lateral point on the widest spot of the same articular facet
CR_OC_	Measured from the most anterior point to the most posterior point of the right occipital condyle	DRS_C1_	Measured on the atlas right superior articular facet from its most anterior point to its most posterior point
CL_OC_	Measured from the most anterior point to the most posterior point of the left occipital condyle	DLS_C1_	Measured on the atlas left superior articular facet from its most anterior point to its most posterior point
E_OC_	Measured from Basion to Opisthion (respectively the most anterior and most posterior point of the foramen magnum)	F_C1_	Measured from the most anterior point to the most posterior point of the vertebral foramen
G_OC_	Measured from the most lateral point on the right side to the most lateral point on the left side of the foramen magnum	H_C1_	Measured from the most lateral point of the right side to the most lateral point of the left side of vertebral foramen
K_OC_	Measured from the most medial points of the right and the left superior articular facets of the occipital condyle	J_C1_	Measured from the most lateral point of the right superior articular facet to the most lateral point of the left superior articular facet
I_OC_	Measured from the most lateral points of the right and left superior articular facets of the occipital condyle	L_C1_	Measured from the most medial points of the right and left superior articular facets

#### 2.1.2 Features extraction and classification models

A classification model was obtained upon verification of repeatability of all measurements: the intra‐ and inter‐observer agreement was calculated by means of the technical error of measurements (TEM). TEM is an accuracy index commonly used in anthropometry to verify the accuracy of repeated anthropometrical measurements when performed by the same and other observers (Perini et al., 2005). The degree of intra‐observer and inter‐observer measurement variation was deemed as acceptable for values ≤7.5% according to Bartlett and Frost (2008) and Arroyo et al. (2010).

The starting database included 150 individuals × 16 measurements (eight for the occipital bone (OC) and eight for the first cervical vertebrae (C1)). From the 16 linear measurements, eight features were extracted, each quantifying the difference between corresponding dimensions in OC and C1: for each *i*th pair of measures *m*, each feature was computed as *m*_C1_ − *m*_OC_, as in Figure 1. A ninth feature was obtained as the difference between the norm of the vectors combining all the linear measurements of C1 and OC, respectively. For instance, the norm of the *n*th OC array was computed as: ${\mathrm{norm}}_{n,\mathrm{OC}}=\sqrt{\sum_{i=1}^{8}m_{i}^{2}}$.

The working database was artificially composed by creating new examples by combining each set of OC measurements with all the others on C1. In other words, we created a new dataset containing all the possible differences of OC with all the available C1, with a total of 150^2^ = 22,500 examples. Of them, 150 were labeled as belonging from “correspondent” pairs and the remaining pairs as “non‐correspondent”.

Six supervised machine learning techniques were trained on this dataset with the Classification Learner tool in Matlab (v. 2018b, The Mathworks Inc., Natwick, USA) to automatically associate the belonging of two remains to the same skeleton. The different methods were chosen to test a spectrum of both linear and nonlinear classification algorithms:

1. Multiple linear discriminant analysis.
2. Logistic regression.
3. Quadratic discriminant.
4. Support vector machine (SVM), which sets hyperplanes defining decision boundaries in a multidimensional space. A Gaussian Kernel was implemented.
5. Boosted trees: classification models are structured as a tree built top‐down from a root node and involves partitioning data into subsets that contain common features based on the decrease in entropy after a dataset is separated. Boosted trees are an extension of decision trees aggregating multiple decision trees into a single result. The number of learners (trees) set in this study was 50.
6. Neural networks: a feedforward network consisting of an input, a hidden and an output layer was designed. Neurons (*n* = 50) in the hidden layer processed the input features according to hyperbolic tangent sigmoid functions. The output layer is a single neuron which returns the predicted class. The backpropagation learning algorithm was used to update the weights and biases of the network. Input data was split into three subsets: 70% for training, 15% for testing, and 15% for validation.

The data processing flow including the main step is represented in Figure 1.

#### 2.1.3 Model validation and statistics

To evaluate the classification accuracy, the models 1–6 underwent a 10‐fold cross‐validation procedure: data were randomly partitioned into 10 sets, and nine of these were used to develop a new model and to evaluate its predictive accuracy using data from the remaining part. This was repeated 10 times, taking the mean performance as the unbiased estimate of the model for the complete dataset. Classifiers were evaluated in terms of sensitivity, specificity, area under curve (the receiver operating characteristic, ROC) (AUC), and, as the dataset was inherently skewed (the non‐correspondent examples outnumbered the correspondent ones by a 150 factor), we computed the precision, namely the positive predictive value (PPV) as:

$$\mathrm{PPV}=\frac{\text{number of true positives}}{\text{number of true positives}+\text{number of false positives}}.$$

Features were presented in terms of mean, SD, confidence interval at 95% (95% CI). Linear Pearson's correlation coefficient (*r*) and the coefficient of determination (*R* ^2^) were computed between correspondent linear measurements. According to Taylor (1990) a correlation of *r* < 0.03 was considered poor, low if 0.3 < *r* ≤ 0.5, moderate if 0.5 < *r* ≤ 0.7 and strong if *r* > 0.7.

### 2.2 3D articular surface survey

A sub‐sample of cranium‐atlas joint set was randomly selected from 46 known individuals (26 females and 20 males with an average age of 60 ± 19 years old) of the overall study sample in order to carry out the 3D analysis on the articular surfaces congruency. A total of 306 superimpositions were generating using match and mismatch combinations.

Two different protocols were used for the acquisition of 3D models of crania and atlases (in.stl format): the 3D models of crania were acquired through a stereophotogrammetric system (Vectra M‐3, Canfield Scientific, Fairfield, NJ, USA), while 3D models of atlases were acquired using a laser scanner system (Dental Wings series 3, Dental Wings Inc., Montreal, Canada). The adoption of two different 3D systems depended on the different dimensions and the dissimilar morphological asset of the two anatomical structures analyzed. More specifically, the laser scanner was the most accurate of the two instruments though limited to measuring the atlas because the instrument was designed for dentition. On the other hand, the stereophotogrammetry system scans large objects, such as the cranium, with satisfactory accuracy but was unsuitable for scanning the atlas because of the complex protocol necessary to position the bone for scanning. Consequently, the atlas and skull were scanned by the two different instruments; yet produced compatible 3D models appropriate for comparison as suggested by a previous study (Codari et al., 2015) which found no significant differences between measurements and superimpositions implemented on/and between 3D surfaces produced by Vectra M‐3 and Dental Wings Series 3.

**FIGURE 2:**
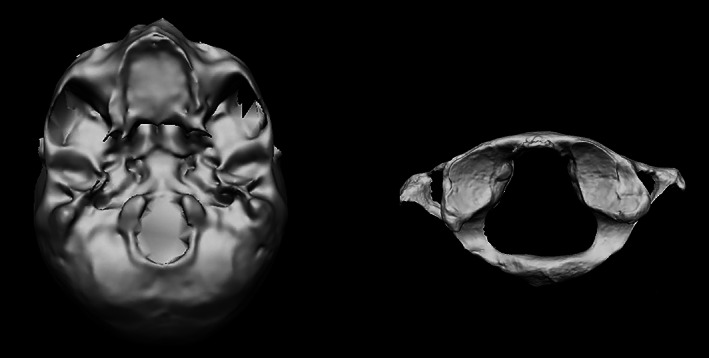
Example of the 3D bone models. Right: Inferior face of the cranium obtained with stereophotogrammetry acquisition. Left: Superior face of atlas obtained with a laser scanning device

Selection of the regions of interest (ROIs) from the 3D models followed an initial acquisition of the 3D models of the crania (obtained by orienting the inferior surface of the cranium toward the objectives of the stereophotogrammetric camera) and the atlas (with the superior face facing the light source) (Figure 2). In this specific case, ROIs corresponded to the entire surface of the two occipital condyles and the two superior articular facets of the atlas.

**FIGURE 3:**
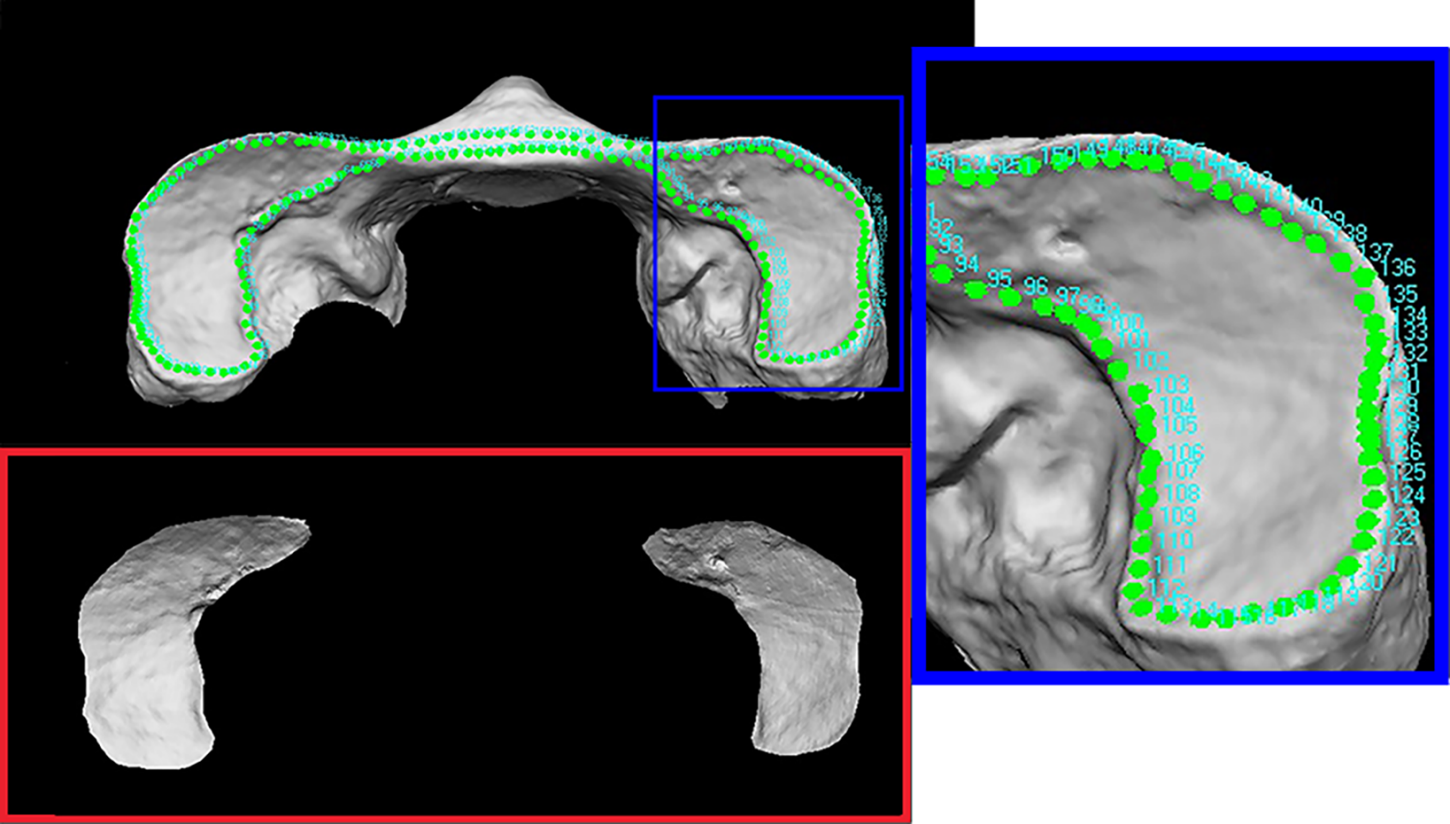
Selection of the ROI from the superior face of atlas obtained with the VAM software. In the blue panel is depicted the over 200 points positioned in the articular surface contour in order to select the area of interest. The red panel shows the final representation of the selected area of interest: The superior articular surfaces of atlas remaining in their original distance and 3D orientation

**FIGURE 4:**
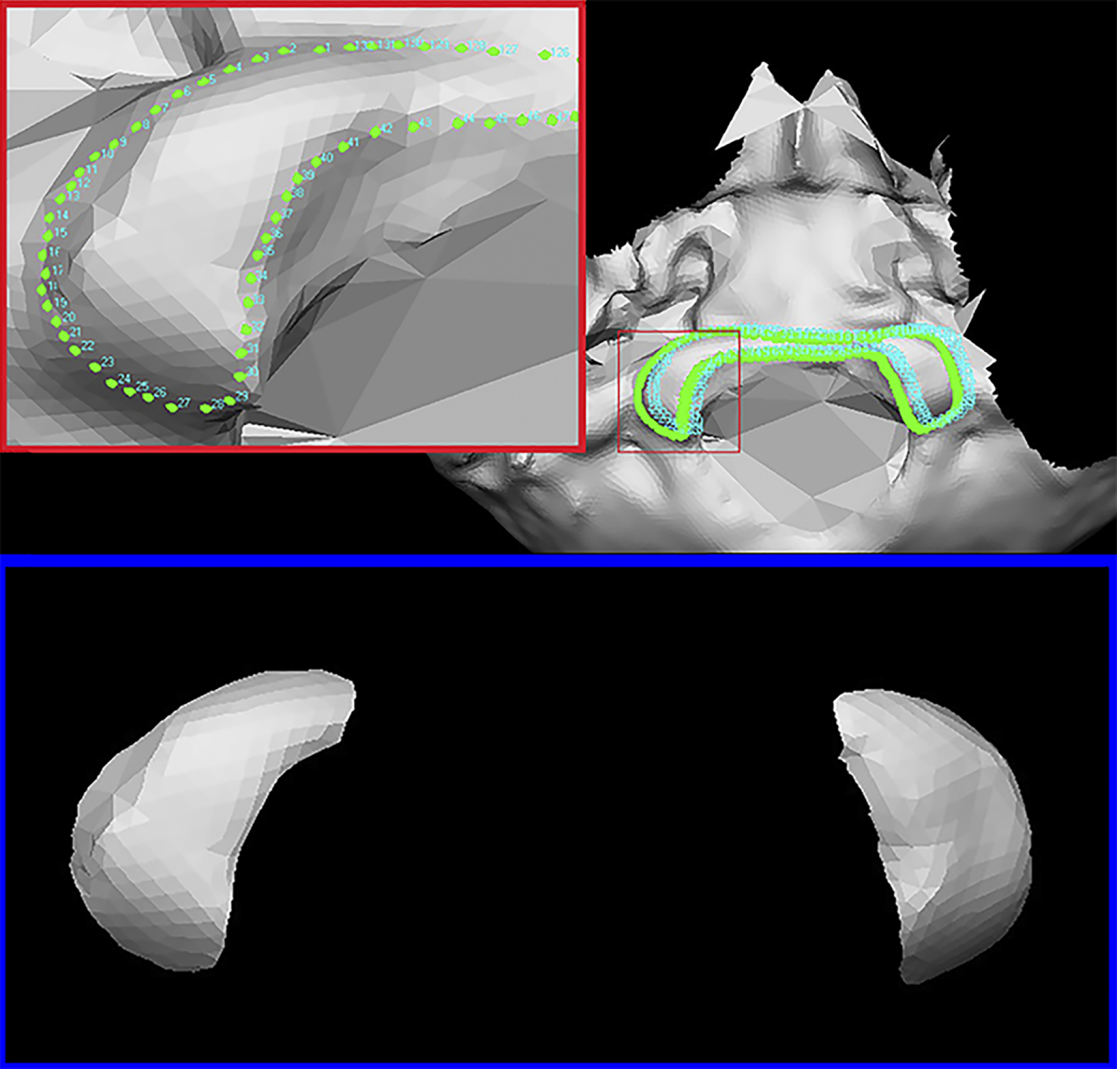
Selection of the ROI from occipital obtained with the VAM software. In the red panel is depicted the numerous points identified on the articular surface contour of the occipital condyles in order to select the area of interest. In the red panel is the final representation of the selected area of interest: The two occipital condylar surfaces remaining in their original distance and 3D orientation

The selection of ROIs was entirely performed through the VAM software (version 2.8.3; Canfield Scientific Inc., Fairfield, NJ, USA), which enabled the analyst to manipulate and process the 3D images to then perform superimposition between two selected ROIs structures. ROIs were selected through the automatically (removed ally) removal of the whole bone surface around the joint surfaces. This was accomplished by manually selecting numerous points positioned on the contours of the articular facets of each 3D model. The same procedure was carried out for both bone elements in order to register 46 3D‐OC (occipital condyles pair models) and 46 3D‐C1 (atlas superior articular facet pair models) ROIs models. In these perspectives, the two occipital condyles (OC) as well as the two superior articular facets of atlas (C1) in each single 3D model maintained the real distances and 3D asset (Figures 3 and 4).

Once ROIs were semi‐automatically selected from each 3D model, the 3D superimposition analysis was conducted according to previous studies (Cappella et al., 2019; Gibelli et al., 2019). The protocol of superimpositions was based on the registration of the 3D scan of both matching ROIs (OC and C1 corresponding to bones from the same individual, namely corresponding OC‐C1) and mismatch ROIs (OC and C1 corresponding to bones deriving from two diverse individuals, namely non‐corresponding OC and C1). An initial correct orientation of OC on C1 models was reached through a landmark‐based registration: in detail, four points were placed on the anterior, posterior, lateral and medial edge of the articular facets and a registration was performed according to the least point‐to‐point distance between the corresponding landmarks. Then the software was requested to perform a novel automatic registration accordingly to the least point‐to‐point distance between the two 3D superimposed entire surfaces (ROIs). Color maps of distances (chromatic representation of areas best fitting or most distant between the two models) and point‐to‐point distances expressed in Root Mean Square (RMS) were automatically calculated for each of the surface pairs by the VAM software. In addition, also the minimum and maximum values of RMS and the SD (expressed in mm) were calculated for each superimposition. A total of 306 superimpositions were performed by the same observer: 46 superimpositions were performed between the scans of atlas and occipital condyles belonging to the same individuals (matches) and 260 superimpositions were performed by using the 3D scans of atlas and occipital condyles belonging to different individuals (130 combinations of atlases and crania from females and 130 combinations from males) in order to produce mismatch superimpositions.

Intra‐ and inter‐observer error was tested in order to evaluate repeatability of the entire 3D protocol and analysis. The technical error of measurement (TEM) was calculated as already described for the linear measurements.

A two‐ways analysis of variance (ANOVA) test was conducted in order to evaluate statistically significant differences between sexes, and between the match and mismatch group. Prior to the two‐ways ANOVA test, the normality and the homoscedasticity of all variables was evaluated using Levene and Jarque‐Bera test respectively (Field, 2009). Finally, differences between the means and the SDs of the two groups were verified. All statistical analysis concerning the 3D survey was performed with SPSS software (version 25.0, SPSS Inc., Chicago, IL, USA). A statistical significance (alpha) level of 0.05 was implemented throughout.

## 3 RESULTS

### 3.1 Linear osteometric survey

Data concerning mean values, SD and relevant 95% confidence interval (CI) are reported for each linear measurement in Table 2 with intra‐ and inter‐observer error. Each measurement proved acceptable and all relative TEM (rTEM) values ranged from 1.8% to 6.8%.

**TABLE 2:** Descriptive statistics (mean, SD and 95% confidence intervals, expressed in mm) of each linear osteometric dimension and related intra‐ and inter‐observer error

Measurements	Mean (SD)	95% CI	Intra‐observer error (%TEM)	Inter‐observer error (%TEM)
AR_OC_	10.5 (1.3)	10.3–10.8	5.6	6.2
AL_OC_	10.9 (1.3)	10.7–11.1	5.8	4.4
CR_OC_	22.6 (2.3)	16.7–29.2	3.9	4.3
CL_OC_	22.6 (2.3)	22.2–23.0	4.3	4.4
E_OC_	31.0 (2.8)	18.6–37.5	2.6	2.7
G_OC_	28.8 (3.4)	28.3–29.4	3.9	3.1
I_OC_	50.5 (4.7)	49.7–51.3	2.9	3.7
K_OC_	16.0 (2.2)	16.0–16.7	5.0	5.4
BRS_C1_	11.3 (1.6)	11.0–11.5	4.0	4.3
BLS_C1_	11.2 (1.6)	10.9–11.5	6.8	5.4
DRS_C1_	23.3 (2.8)	22.9–23.8	3.5	5.5
DLS_C1_	23.4 (2.7)	22.9–23.9	6.0	2.4
F_C1_	35.4 (2.8)	34.9–35.8	1.8	5.0
H_C1_	30.3 (2.4)	30.0–30.7	2.9	1.9
J_C1_	50.0 (4.1)	49.3–50.6	2.0	2.4
L_C1_	16.0 (2.1)	15.6–16.3	4.0	5.3

Abbreviations: see Table 1 for definitions.

**FIGURE 5:**
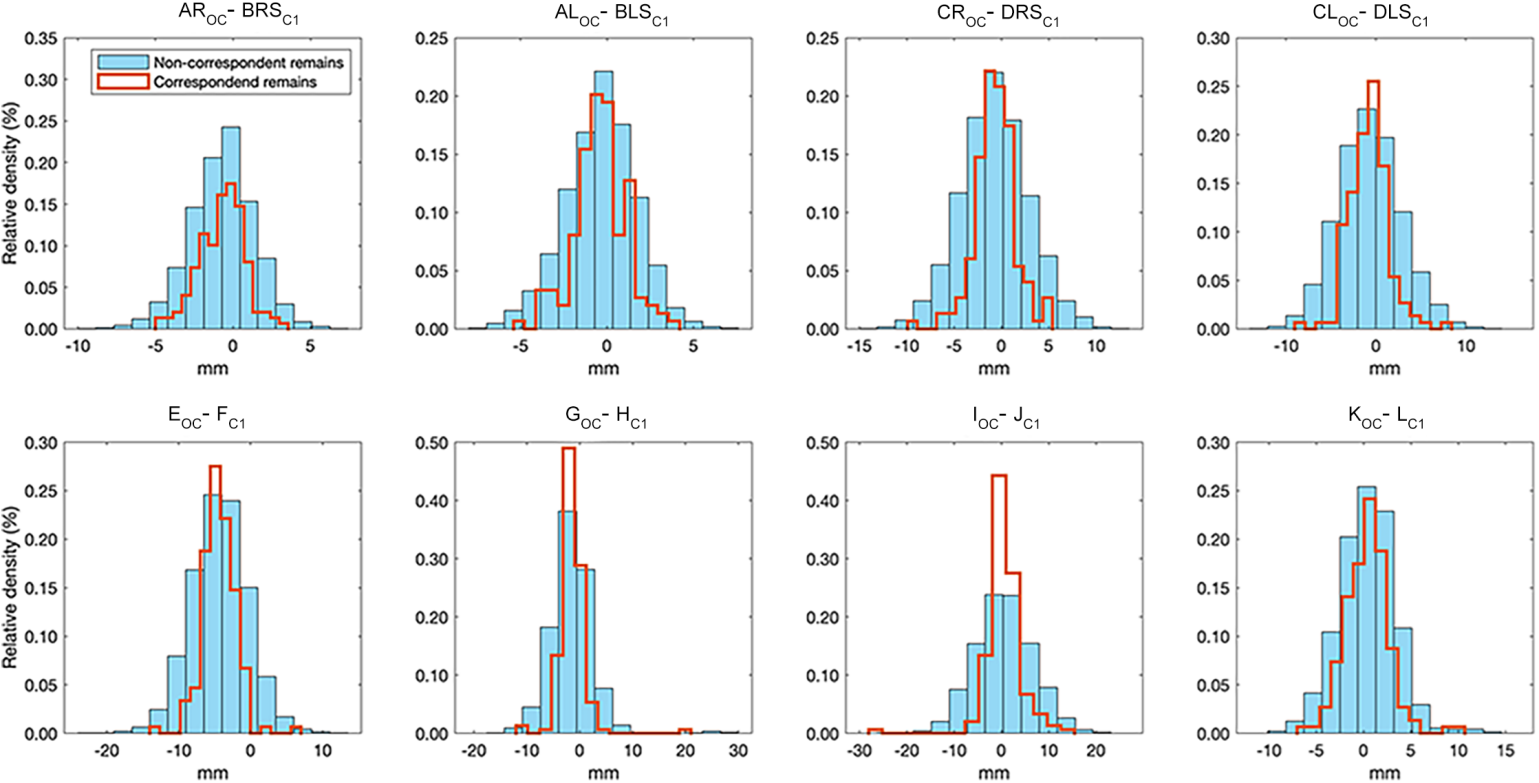
Distribution of linear‐based features for the correspondent pairs (red line), compared with the examples of non‐correspondent pairs (shaded bars)

Feature values for correspondent pair variables are shown in Table 3. The correlation between correspondent measurements reached statistical significance for every pair, ranging from moderate to low, with the exception of the norm of all C1 and OC values. Features distributions of correspondent and non‐correspondent pairs are represented in Figure 5, and the two were almost always overlapped. No classifier was able to correctly detect a single correspondent pair of specimens. Table 4 shows the negative, although informative results of the classification outcome. The high values of specificity should be read in light of the skewed sample.

**TABLE 3:** Feature statistics (mean, SD and 95% confidence intervals, expressed in mm), including Pearson's correlation of correspondent measurements

Feature	Description	Mean (SD)	95% CI		*r*	*R* ^2^	*p*
1	AR_OC_ – BRS_C1_	−0.7 (2.1)	−1.02 –	−0.39	0.514	0.264	<0.001
2	AL_OC_ – BLS _C1_	−0.4 (2.6)	−0.70 –	−0.06	0.481	0.231	<0.001
3	CR_OC_ – DRS _C1_	−0.7 (3.6)	−1.13 –	−0.21	0.650	0.443	<0.001
4	CL_OC_ – DLS _C1_	−0.73 (3.5)	−1.20 –	−0.25	0.622	0.387	<0.001
5	E_OC_ – F _C1_	−4.38 (3.9)	−4.86 –	−3.89	0.671	0.449	<0.001
6	G_OC_ – H _C1_	−1.50 (4.2)	−2.10 –	−0.91	0.591	0.349	<0.001
7	I_OC_ – J _C1_	0.59 (6.2)	−0.26 –	1.43	0.606	0.367	<0.001
8	K_OC_ – L _C1_	0.35 (3.0)	−0.15 –	0.85	0.402	0.161	<0.001
9	norm_OC_ – norm_C1_	−2.6 (2.7)	−3.03 –	−2.07	0.838	0.702	<0.001

Abbreviations: See Table 1 for definitions; *r*, linear Pearson's correlation coefficient; *R* ^2^, coefficient of determination; CI, confidence interval; SD, standard deviation.

**TABLE 4:** Classification performance of machine learning models

Classifier	PPV	Sensitivity (%)	Specificity (%)	AUC
Linear discriminant	n.a.	n.a.	0.99	0.40
Logistic regression	n.a.	n.a.	0.99	0.40
Quadratic discriminant	0	0	0.99	0.81
SVM	n.a.	n.a.	0.99	0.79
Boosted trees	n.a.	n.a.	0.99	0.79
Neural network	0	n.a.	0.99	0.52

Abbreviations: AUC, area under curve; SVM, super vector machine; PPV, positive predictive value.

### 3.2 3D articular surfaces survey

Intra‐ and inter‐observer error proved acceptable values for all groups, as reported in Table 5.

**TABLE 5:** Intra‐ and inter‐observer error expressed in absolute (TEM) and relative (rTEM)

	Intra‐observer error		Inter‐observer error	
	Matches	Mismatches	Matches	Mismatches
TEM (mm)	0.021	0.029	0.018	0.032
rTEM (%)	6.8	5.7	5.7	6.1

Abbreviations: rTEM, relative technical error of measurement; TEM, technical error of measurement.

RMS, minimum, and maximum values and SD of ‘match’ and ‘mismatch’ superimpositions are summarized in Table 6 for both sexes. All the values in different groups were found normally distributed and homoscedastic (*p* > 0.05). No statistical differences between the sexes were found (*F*: 2.36; *p*: 0.125): nevertheless, RMS values were significantly different among ‘match’ and ‘mismatch’ group (*F*: 22.65; *p* < 0.0001).

**TABLE 6:** Maximum, minimum, mean RMS and SD for matches and mismatches in males and females

	*N*	Matches (mm)	*N*	Mismatches (mm)
Males				
Mean	20	0.28	130	0.51
SD		0.08		0.22
Max		0.51		1.49
Min		0.16		0.18
Females				
Mean	26	0.31	130	0.41
SD		0.11		0.17
Max		0.51		1.01
Min		0.15	260	0.14
Total				
Mean		0.29		0.46
SD		0.09		0.20
Max		0.53		1.49
Min		0.15		0.14

*Note*: Min and max refer, respectively, to the mean minimum RMS and maximum RMS of each group.

Abbreviations: mm, millimeters; Max, maximum RMS value; Min, minimum RMS value; *N*, number of cases; SD, standard deviation.

**FIGURE 6:**
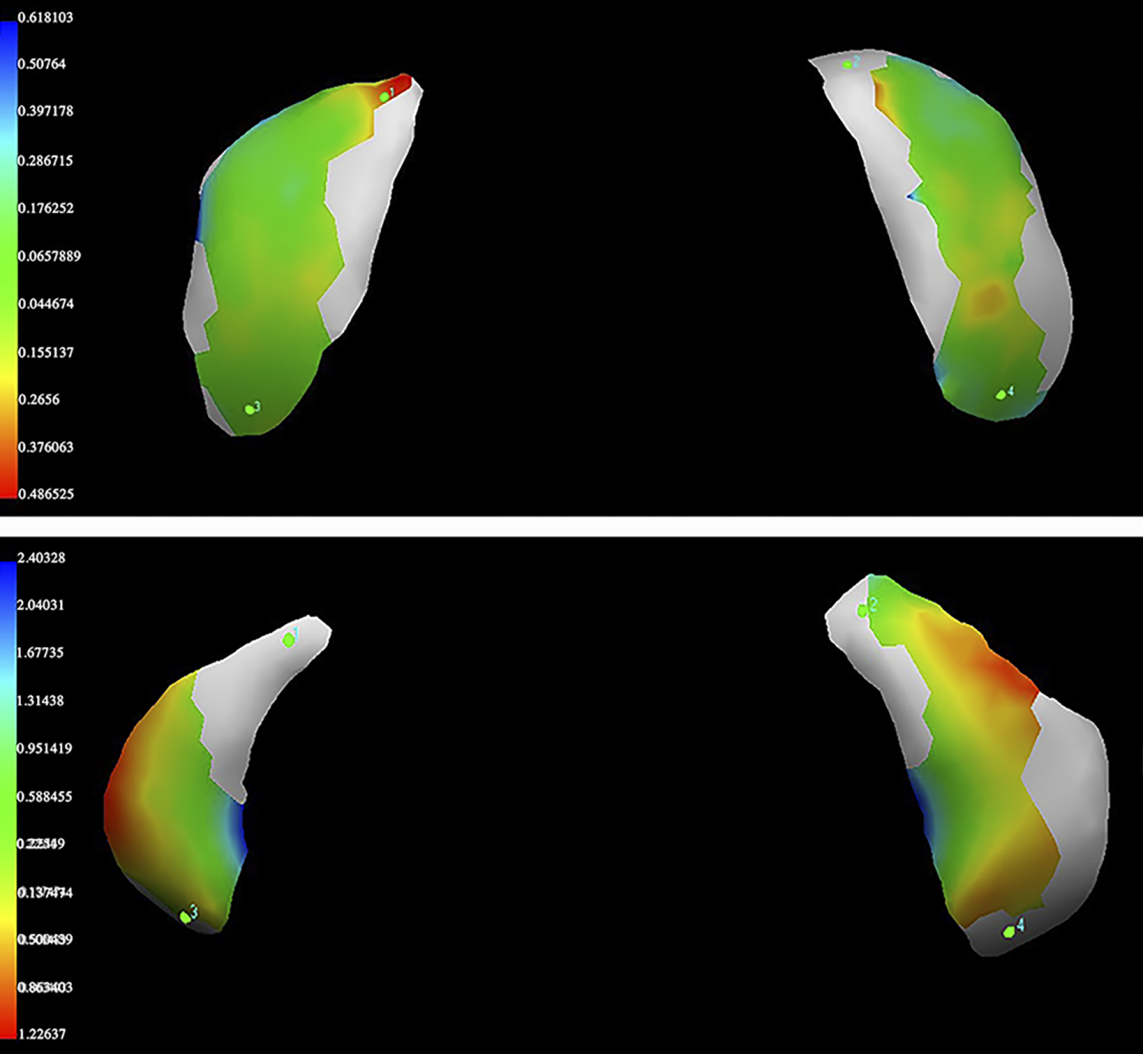
Examples of two superimpositions between corresponding atlas‐occipital condyles (upward) and non‐corresponding atlas‐occipital condyles (beneath). The color map offers an initial and easy representation of the point‐to‐point distances: Green represents well superimposable areas with point‐to‐point distances near 0; red and blue represent areas and points not well superimposed and with greater distances. The superimposed area of the matching atlas‐occipital condyles is mostly colored in green (with the RMS value corresponding to 0.20 mm), while the superimposed area of the mismatching atlas‐occipital condyles is colored mostly in red and yellow proving so higher distances, as also confirmed by the RMS value (0.94 mm)

**FIGURE 7:**
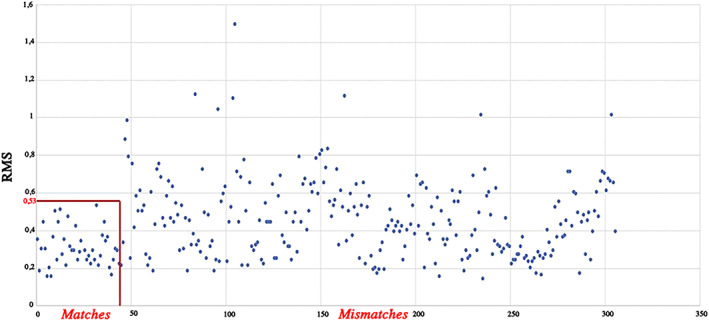
Scatter plot of RMS values of each ‘matches’ and ‘mismatches’ superimposition. The red box indicates the RMS values of all superimpositions of the ‘match’ group, always lower that the threshold value of 0.53 mm. RMS values pertaining the superimposition of the ‘mismatch’ group are distributed in the range between 0.14 and 1.18 mm with 68% of the values lower than the threshold value of matches

On average, RMS values ranged between 0.14 and 0.53 mm in ‘matches’ and between 0.14 and 1.49 mm in ‘mismatches’ (Figures 6 and 7). Overall, all ‘matches’ superimpositions showed RMS values always lower than 0.53 mm with 63% of RMS values ranging between 0.14 and 0.30 mm and 20% ranging between 0.31 and 0.40 mm. On the contrary, ‘mismatch’ superimpositions were equally distributed across a span of RMS values ranging from 0.14 to 1.49 mm with 32% of RMS values higher than 0.53 mm which is the maximum RMS value found in the match group (Figure 7). In these terms, among the total of 306 superimpositions including both ‘matches’ and ‘mismatches’, only the latter generated values over 0.53 mm and thus this value can be considered as the overall threshold for excluding incorrect matching (one third of the ‘mismatch’ superimpositions).

## 4 DISCUSSION

The analysis of commingled human remains is a complex process that requires a critical and systematic approach and the use of reliable techniques (Rodríguez et al., 2016). Commingled contexts can be represented by various scenarios: from bodies completely skeletonized and chaotically disarticulated to corpses scarcely skeletonized and only partially disarticulated. Indeed, sometimes the disarticulation can involve many body parts while other times just few or a single skeletal element. Although the latter could appear to be an easier scenario, it is still particularly critical: if two bones can be re‐associated together entailing also the association of the entire body still articulated with one of the two bones, then the final result will be the correct association of the entire body, allowing one to accomplish important legal, ethical and/or forensic duties. This is the case of the atlanto‐occipital joint, which can permit the re‐association of the cranium to the entire body if the disarticulation is limited only to this specific joint. Despite the importance such a joint can demonstrate for the re‐association of crania and post‐cranial skeletons in commingled contexts, this articulation still needs an in‐depth examination for this specific anthropological purpose. Consequently, the approaches presented in this study comprise two different analyses: an osteometric and a 3Dsurface analysis of the occipital bone (OC) and atlas as well as an examination of the difficulties in finding morphological and metric congruencies between the two articulating bones.

### 4.1 Reproducibility of linear measurements

One of the first aspects to consider prior to discussing the results concerning the 2D and 3D analysis is the reproducibility and repeatability of such approaches. The linear measurements tested by the present study represented the starting point to build a possible statistical classification potentially able to correctly classify corresponding and non‐corresponding bones. The 16 linear metric parameters tested by the current study proved to be repeatable and reproducible: the intra‐ and inter‐observer errors proved acceptable with a rTEM range of 1.8%–6.8%. These results are consistent with what was reported by Dudar and Castillo (2016). However, a high variation of rTEM values was observed for both intra‐ and inter‐observer error. The higher values can be related to difficulties in measuring some poorly defined anatomical points. For instance, some measurements might be more difficult and subjected to a larger margin of error since the articular surface area is more irregular and/or less pronounced, an issue which can possibly explain the high variability of rTEM. According to a previous study (Kouchi et al., 1999), even when the definitions of anatomical landmarks and the measurement descriptions are clear, the ambiguity in the practical procedures (in locating landmarks and in using the instruments) can lead the observers to develop their own measurement technique causing inter‐observer errors. Nevertheless, despite the high variability, all rTEMs proved acceptable thus allowing the use of all tested measurements for the further statistical analysis: the extraction of the features and the creation of a potential model classification.

### 4.2 Classification models based on linear measurements: Focal points and problems

Several approaches were used in order to create a potential classificatory model based on osteometric pair variables of the atlas and cranium: multiple linear discriminant analysis, logistic regression, quadratic discriminant, support vector machine, boosted trees and neural networks. A relevant finding of this study is that the automatic re‐association of crania and atlases based on linear metric measurements is hardly viable, even if sophisticated classification models based on machine learning techniques are used. The differences between measurements of the articular facets of corresponding bones from the same individual are not so exclusive as to permit their re‐association when recovered from commingled contexts (Figure 1).

This result is not surprising given the feature distributions for correspondent and non‐correspondent remains (Figure 5), where a number of atlases were a better match based on linear dimensions for a cranium than the corresponding atlas. Consequently, linear dimensions alone are not sufficiently descriptive of the geometry and characteristics of two corresponding specimens to allow for a straightforward association. This is reinforced by the weak‐to‐moderate correlation between correspondent dimensions (Table 3). One possible explanation is the high variability in all features considered, represented by the SD compared to the respective mean values. The high variability of the considered metric features and, consequently, some high SD values, might be due to the absence of the cartilage normally existing between the occipital condyles and the articular facets of the atlas: such structures together with the synovial ones are all present ‘in vivo’ and participate all together in the joint allowing its movements as well as the compensation of possible differences and normal incongruencies between the two corresponding articular structures. In addition, other soft tissue structures should be considered as the ligaments and the muscles that together contribute to the stabilization and functionality of the atlanto‐occipital joint. The absence of such components might be the reason for the mismatch between the two articular surfaces and hence the significant differences on the measurements taken on dry bones. A similar explanation was reported by Preissler et al. (2018) who found incongruencies between corresponding measurements of mandible condyles and fossa mandibulari which they interpreted as partly arising from the anatomy and function of such an articulation. By extension, the functional and anatomical properties of the atlanto‐occipital must be considered: overall, the joint has an atypical morphological conformation in humans as it exhibits a convex‐concave configuration with the articular facets being slightly more curved in the medial‐lateral direction. This peculiar joint has the role to provide sufficient mobility of the head while allowing adequate stability to protect the spinal cord and support the head. In particular, the occipital condyles have a convex curvature while the superior facets of the atlas have an associated concavity: such a configuration allows motion in the frontal plane which determines flexion and extension. Furthermore, the pronounced anterior to posterior convexity of the articular complex allows for minor motion in the sagittal plane (Cattrysse et al., 2016). Consequently, the morphological configuration of the condylar‐atlas joint results in a lack of plasticity while maintaining a sufficient range of motion, which can subsequently vary among individuals. Variation of facet angulation and morphology as well as the specific ligament configuration that stabilizes this joint may affect motion of the joint, determining more ample or restricted movements in specific directions. The variation in motion is the consequence of joint configuration which can be partly explained by the morphological variability of the involved articular facets (Cattrysse et al., 2016) which may be related to inter‐individual variability still to be quantified.

Whether or not there are sufficient differences in joint morphology between individuals to allow differentiation has not yet been demonstrated, though some degree of motion range has been reported in literature for the atlanto‐occipital joint (Chancey et al., 2007; Siccardi et al., 2020). Indeed, the articular surfaces of this joint have a large (important) inter‐ and intra‐individual variation as proven by several studies. Naderi et al. (2005) reported data concerning the morphological variation of occipital condyles in a Turkish population and found that although the ‘oval’ type is the most frequent (50% of individuals) numerous other shapes were reported with minor frequencies. Dudar and Castillo (2016) analyzed the articular facets of the atlas in addition to the occipital condyles and reported significant differences on their shape between sexes and subgroups of different ethnicity. Furthermore, they found that in each individual the shape match between the right and left side reaches percentages slightly higher than 50%, and that there is a very poor shape correspondence between occipital condyles and atlas facets at both individual and inter‐individual levels. Overall, the reported results indicate that multifactorial genetic and environmental differences of different populations are expressed in the morphology of the atlanto‐occipital joint and need to be verified in specific reference populations (Dudar and Castillo, 2016). Given the reported shape incongruency for the occipital condyles, we decided to focus only on osteometry and the 3D geometrical configuration for investigating the aim of our study. The morphometric variation of this joint among individuals and populations has been in fact already argued in the literature. Regarding the overall articulation, Dudar and Castillo (2016) found that among the linear measurements examined in the two articular surfaces, the delta variation of five linear distances (antero‐posterior length and medio‐lateral width of the articular surfaces, minimum and maximum breadth of the foramen magnum and vertebral foramen and antero‐posterior and medio‐lateral diameter of the foramen magnum and vertebral foramen) and three derived measurements (surface areas, radii and curvatures of the occipital condyles and atlas facets) displayed congruency and significant sexual and ancestry effect, proving the existence of a quantitative anatomical variability among individuals. Briggs et al. (2008) reported asymmetry and absence of exactness of fit between corresponding joint surfaces of the occipital condyles and superior facets of the atlas: they found an overlap between measurements of the surfaces greater than 2 mm in 10 out of the 12 articulations analyzed in their study. However the study was conducted by performing photometric and point counting analysis (indirect 2D techniques) on a very limited numbers of cadaveric joints fixed in formaldehyde, and thus the data were obtained by performing techniques different from the osteometric techniques conducted directly on dry bones, making difficult any comparison with differently designed studies. In these terms, although the qualitative and metric congruency between paired measurements on the superior articular facets of the atlas and the occipital condyles of the cranium was found to be scarce at times, the metric variation identified among individuals of different sex and ethnicity led us to test if such variation has individualizing potential useful for the re‐association of these two adjoining bones.

Of utmost importance for many scholars is the practical reason behind the need to re‐associate the cranium with the atlas, with the rest of the body if the disarticulation concerns uniquely this joint. It is important to find a method able to re‐associate these two bones in order to apply it to a recent mass disaster presenting a commingled context where part of the commingled remains are represented by entire decomposing corpses with the crania disarticulated (Cattaneo et al., 2020). Finding a feasible method for this purpose would allow the entire victims body (remains) to be assembled and the anthropological analysis to progress, thus aiding the identification process.

Our attempt of building a method based solely on osteometric parameters for the re‐association of such a joint proved to fail even if advanced automatic statistical approaches were performed, highlighting the unfeasibility to rely on corresponding linear distances for the re‐association of the bones joining at this specific articulation, and the absence of an exclusive and individualizing congruency in agreement with the conclusions reported by Dudar and Castillo (2016). Notwithstanding the results of our osteometric survey, according to Ubelaker (2008) and Byrd and Adams (2003) several studies reported the possibility of sorting human skeletal remains through osteometry although with reservations. However, the types of joints, the bone elements considered, the type of approach (if pair matching or articulating bone) as well as the diverse statistical approaches used are crucial factors responsible for differences among results from different studies. For instance, Buikstra and Gordon (1980) proposed a metric method for assessing probability that two vertebrae belong to the same individual: a series of measurements were taken on the vertebral foramen and body and a statistical model for testing congruence between adjacent elements was developed. Although an objective method was described, their findings, similarly to our results, showed a poor congruence between adjacent elements concluding that the size of the body of cervical vertebrae is not sufficient for the re‐association but helps to minimize underestimating the true minimal number of remains present. London and Curran (1986) and London and Hunt (1998) tried to re‐associate the hip joint in commingled skeletal remains and found a significant correlation between the femur head diameters and the related measurements of acetabulum. However, in a follow‐up study London and Hunt (1998) proposed that osteometric sorting of the hip joint should be always supported by visual re‐association.

Adams and Byrd (2005, 2008) developed several regression models and converted the metric data into a natural logarithm, which predicts the dependent variable from the independent one. The independent variable is represented by one of the metric measurements of the sample and is entered into the regression model formula to produce a predicted value for the other bone measurement (the dependent variable). Thus, whether the measurement value of the bone specimen falls within the prediction interval surrounding the predicted value, the null hypothesis (that two bones originating from the same individual) is accepted. In addition, the combination of multiple measurements (mostly pertaining to long bones), namely their summation, proved to lead to a significantly higher correlation between two bones: the linear osteometric measurements used as variables in their analysis have shown a correlation coefficient of 0.80 or higher. In particular, one study (Rodriguez et al., 2016) confirmed relevant results when the osteometric sorting methods proposed by Adams and Byrd (2005, 2008) were tested on simulated commingled remains of a Colombian population. In addition, concerning again the measurements of long bones, Byrd and LeGarde (2018) demonstrated that the correlation coefficient of the regression models was higher when bone lengths were used. However, other authors suggested that these proposed methods allow too many false rejections when used for predicting pair matching, undermining the ability of such approaches in showing incompatibilities for potential matching (Vickers et al., 2015).

Although these studies reported positive findings and the real possibility of sorting bones through osteometric analysis, the approaches were mostly focused on pair matching of bone elements rather than the re‐association of matching elements at the joint, the latter being the objective of the present study. Investigations on sorting by articulating bone portions are scarce and limited to few joint types, such as the talus and calcaneus (Anastopoulou et al., 2018) mandible and cranium (Preissler et al., 2018), and hip joint (London & Curran, 1986; London & Hunt, 1998). Overall, the type of articulation and the related functional anatomy may be possible factors behind the differences in the outcomes and reliability of re‐associating adjoining bones of different anatomical districts through osteometry. In particular, joint surfaces, such as those of the hip examined by London and Hunt (1998) and the talus and calcaneus analyzed by Anastopoulou et al. (2018) demonstrated a higher osteometric congruency, thus providing a greater potential for re‐associating such bones likely because their anatomical function, their linked muscle mass and their repetitive behavior and motion effect the expression of more individualizing anatomy on these joints.

### 4.3 The use of 3D surfaces: Potential and advances

The results so far discussed hold just for linear measurements: nonlinear or volumetric and 3D features might capture additional details that would enable an automatic classification. In fact, when different parameters were examined, as the 3D articular surfaces, slightly more optimistic results were reached. In general, the analysis of the RMS values provides complete information on 3D morphology discordance and quantitative distances as it considers all the distances regardless of which model exceeds the other one, an approach more often applied to forensic purposes (Cappella et al., 2019; Gibelli et al., 2019). In this specific case, the statistically significant differences observed between the groups of ‘matches’ and ‘mismatches’ through the calculation of RMS provided a quantitative parameter for assessing differences between 3D articular facets models (Figure 6). The RMS values of the two groups were useful points of reference allowing the definition of values for partially discriminating between superimpositions of corresponding and non‐corresponding bones. An important threshold value was identified, where the maximum RMS value of the ‘matches’ was 0.53 mm and over this value only ‘mismatches’ were found. This approach is therefore a potential exclusionary sorting method for corresponding bones at this joint. While RMS values of the ‘mismatches’ are equally distributed between 0.14 and 1.49 mm, with the higher percentage (68%) in the same range as the ‘match’ group, no superimpositions of corresponding bones were found over the threshold value of 0.53 mm. On the other hand, for values lower than 0.53 mm, it is not possible to establish whether the superimposition is between two bones belonging to the same person or to different individuals.

Our study revealed the 3D analysis did not allow for the development of a re‐association method for crania and atlases, as the original intent of the direct osteometric approach, but it provides at least additional information that can be useful for an exclusionary screening process. This approach enabled the analyst to exclude almost 35% of non‐corresponding atlases and crania. Therefore, the 3D analysis should be considered as an exclusion sorting method rather than a re‐association one.

Further parameters for the atlanto‐occipital joint should be considered and combined with the present approach in future studies in order to provide combined approaches based on more satisfactory results. The investigation should not only focus on the anatomical and geometrical configuration as verified by the present study but also on additional individualizing variables such as taphonomy, pathology, and enthesopathy. In fact, the presence of a consistent pathological characteristic on two bones congruent or matchable for similarity is an additional individualizing feature that improves the chance of matching adjoining bones correctly. Thus, whenever possible the conclusion should be supported by multiple lines of evidence: the larger the number of characteristics in common between the paired bones, the higher the likelihood that the two bones belong to the same individual (Adams & Byrd, 2014). Indeed, the visual inspection cannot be limited only to the general morphology and joint congruency but should be extended to patterns such as the continuity of traumatic or pathological and taphonomical features, whenever displayed. However, taphonomic or pathological coherence between two elements is not sufficient alone for the re‐association and cannot be consider per se as a sorting technique. This is to say that the resolution of commingled remains in terms of association of skeletal elements should comprise a plethora of different techniques (visual pair‐matching, osteometric and articulation comparison, and taphonomy and pathological/traumatic pattering) used in conjunction with each other to reach the best success, and so the majority of the sorting, associating, and exclusionary procedures are not stand‐alone techniques.

The anthropological ‘eye’ and experience, namely the visual assessment, might also play an important and additional role and can potentially be combined with the automatic and objective approaches discussed here. This is in agreement with Anastopoulou et al. (2018) who presented several equations in order to re‐associate the talus and calcaneus but despite the positive correlations found for several measurements that the metric method is not applicable as a stand‐alone technique, especially when individuals are similar in size, thus suggesting that the final assessment should be also confirmed by the morphological compatibility of the associated articular facets through a visual approach. Our scanning and digital reconstruction of the articular facets actually resemble the visual assessments made by the analysts, and was intended as a first step toward the definition of computerized protocols. Unfortunately, we had to use two different optical scanners (laser and sterephotogrammetry) as detailed in Section 2, that had already been compared in literature (Codari et al., 2015). Even if the original surface data obtained with either scanner were comparable [or did not differ significantly], this represents a limitation of the study, and can be accepted due to the unique scenario of the Mediterranean Sea mass disaster (Cattaneo et al., 2020).

Finally, DNA analysis is the only way to provide conclusive evidence as to whether bones belong to the same individual, but there are a number of disadvantages: cost, logistic problems, destructive processes, and possible contamination from exogenous sources. DNA analysis should therefore only be performed after possible matches have been identified using techniques such as those reported here.

## 5 CONCLUSION

It is widely accepted how challenging can be to find a perfect congruence in linear and 3D geometry between the articular surface of two articulating bones and in particular between the atlanto‐occipital joint: the ‘in vivo’ anatomy of such structures surely is not perfectly reflected in dry bones, where many of the structures participating in the overall ‘in vivo’ articulation are missing. If on one hand, the results suggest an inability to re‐associate a cranium and atlas with high specificity and sensitivity, especially when only using linear osteometry, on the other hand, the use of 3D tools allowed for the configuration of articular surfaces to identify incorrect re‐associations. The use of such technology should be seen not as a substitute, but as a supplementing tool to be used as a sort of exclusionary screening and in combination with other sorting and re‐association techniques once validated in the future.

One has always to remember that the ideal ‘fit’ between two corresponding bones, in terms of linear osteometrics and 3D geometry, does not necessarily exist. Whichever techniques or combination of techniques are used, genetics should be considered fundamental for confirming the consistency of a possible match, aiding in the final decision of confirming or excluding if the selected skeletal elements are coming from the same individual. Finally, as proved by the present study, the re‐association of some articulations may be extremely difficult if not impossible to determine with certainty when some techniques are used. In these cases, the authors would advise the anthropologists to reason in terms of exclusion rather of re‐association, and to rely on a holistic approach requiring the use of multiple combined techniques or, when not possible, to state the impossibility of making a definitive determination.

## CONFLICT OF INTEREST

The authors have no financial interests or other forms of conflicts of interest.

## AUTHOR CONTRIBUTIONS

**Annalisa Cappella:** Conceptualization (lead); data curation (equal); formal analysis (equal); investigation (lead); methodology (lead); project administration (lead); software (equal); writing – original draft (lead); writing – review and editing (equal). **Luciana Affatato:** Data curation (equal); formal analysis (equal); investigation (supporting); methodology (supporting); software (equal); visualization (supporting); writing – review and editing (supporting). **Daniele Gibelli:** Formal analysis (supporting); investigation (supporting); methodology (supporting); software (supporting); writing – review and editing (supporting). **Debora Mazzarelli:** Formal analysis (supporting); investigation (supporting); methodology (supporting); resources (supporting); writing – review and editing (supporting). **Matteo Zago:** Data curation (supporting); formal analysis (supporting); investigation (supporting); software (supporting); writing – review and editing (supporting). **Claudia Dolci:** Formal analysis (supporting); investigation (supporting); visualization (supporting); writing – review and editing (supporting). **Chiarella Sforza:** Conceptualization (supporting); resources (equal); supervision (equal); writing – review and editing (equal). **Cristina Cattaneo:** Conceptualization (supporting); resources (equal); supervision (equal); writing – review and editing (equal).

## Data Availability

The data that support the findings of this study are available from the corresponding author upon reasonable request.
